# Cryo‐EM structure of fission yeast tetrameric α‐mannosidase Ams1

**DOI:** 10.1002/2211-5463.12988

**Published:** 2020-10-20

**Authors:** Jianxiu Zhang, Ying‐Ying Wang, Li‐Lin Du, Keqiong Ye

**Affiliations:** ^1^ Key Laboratory of RNA Biology CAS Center for Excellence in Biomacromolecules Institute of Biophysics Chinese Academy of Sciences Beijing China; ^2^ University of Chinese Academy of Sciences Beijing China; ^3^ National Institute of Biological Sciences Beijing China; ^4^ Tsinghua Institute of Multidisciplinary Biomedical Research Tsinghua University Beijing China

**Keywords:** catabolism, Cryo‐EM structure, N‐glycoprotein, selective autophagy, α‐mannosidase

## Abstract

Fungal α‐mannosidase Ams1 and its mammalian homolog MAN2C1 hydrolyze terminal α‐linked mannoses in free oligosaccharides released from misfolded glycoproteins or lipid‐linked oligosaccharide donors. Ams1 is transported by selective autophagy into vacuoles. Here, we determine the tetrameric structure of Ams1 from the fission yeast *Schizosaccharomyces pombe* at 3.2 Å resolution by cryo‐electron microscopy. Distinct from a low resolution structure of *S. cerevisiae* Ams1, *S. pombe* Ams1 has a prominent N‐terminal tail that mediates tetramerization and an extra β‐sheet domain. Ams1 shares a conserved active site with other enzymes in glycoside hydrolase family 38, to which Ams1 belongs, but contains extra N‐terminal domains involved in tetramerization. The atomic structure of Ams1 reported here will aid understanding of its enzymatic activity and transport mechanism.

## Graphical abstract

The α‐mannosidase Ams1 is involved in catabolism of oligosaccharides and is transported from the cytoplasm into vacuoles by selective autophagy pathways in yeasts. The cryo‐EM structure of *S. pombe* Ams1 determined at 3.2 Å resolution reveals the detailed interactions mediating tetramerization, the configuration of the active site, and the differences with a low‐resolution structure of *S. cerevisiae* Ams1.

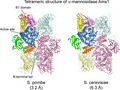

**Abbreviations**
bLAM: bovine lysosomal α‐mannosidase
cryo‐EM: cryo‐electron microscopy
Cvt: cytoplasm‐to‐vacuole targeting
dGMII: Drosophila Golgi α‐mannosidase II
ER: endoplasmic reticulum
FOS: free oligosaccharides
GH38: glycoside hydrolase family 38
JBα‐man: Jack bean α‐mannosidase
N‐glycan: asparagine‐linked glycan
NVT: Nbr1‐mediated vacuole targeting
ScAms1: S.nonbreakingspacecerevisiae Ams1
SpAms1: S.nonbreakingspacepombe Ams1
SpGH38: Streptococcus pyogenes α‐mannosidase

Asparagine‐linked glycans (N‐glycans) are one of the most important modifications in eukaryotic proteins and plays a crucial role in protein folding and quality control and molecular recognition [1, 2]. Biosynthesis of N‐glycans starts with the assembly of a donor substrate dolicholpyrophosphate‐linked Glc_3_Man_9_GlcNAc_2_ (Glc: glucose; Man: mannose; GlcNAc: N‐acetylglucosamine) on the membrane of the endoplasmic reticulum (ER). The lipid‐linked oligosaccharide is transferred to selected asparagine residues in nascent polypeptides by the oligosaccharyltransferase. N‐glycans are then trimmed by glycosidases and extended by glycosyltransferases in the ER and Golgi, giving rise to their myriad structures.

Catabolism of glycoproteins is important for maintaining cellular homeostasis. Most N‐glycoproteins are degraded in the lysosome/vacuole [3], where N‐glycans are digested by exoglycosidases at the nonreducing end and released from peptides with cleavage of the glycan‐peptide bond at the reducing end. In addition, nascent glycoproteins that do not correctly fold in the ER are targeted for ER‐associated degradation, a process in which misfolded glycoproteins are recognized by carbohydrate‐binding lectins and retrotranslocated to the cytosol for proteasomal degradation [2, 4].

In the cytosol of *Saccharomyces cerevisiae*, free oligosaccharides (FOSs) are released from misfolded and retrotranslocated glycoproteins by the cytosolic peptide:N‐glycanase Png1 [5, 6]. In addition, a minor amount of FOSs are produced by oligosaccharyltransferase‐mediated hydrolysis of lipid‐linked oligosaccharides in the ER lumen and translocated to the cytosol [7]. FOSs in the cytosol are digested by the α‐mannosidase Ams1 into the end product Man_1_GlcNAc_2_ [5, 8, 9, 10]. In mammalians, FOSs released from lipid‐linked oligosaccharides and misfolded glycoproteins are digested by cytosolic α‐mannosidase MAN2C1 into a Man_5_GlcNAc isomer that is transported and degraded in lysosomes [4, 11, 12]. Interestingly, MAN2C1 also has functions beyond oligosaccharide catabolism, as it was shown to modulate apoptosis independently of its enzymatic activity[13] and promote tumorigenesis by binding to and inhibiting the PTEN phosphatase [14].

Ams1 and MAN2C1 are homologous proteins and belong to glycoside hydrolase family 38 (GH38) that hydrolyzes terminal α‐linked mannose with net retention of anomeric configuration [15, 16]. They are also classified as subtype C of Class II mannosidase [17]. Enzymes in the GH38 family are involved in biosynthesis and degradation of N‐glycans. Structurally, Golgi α‐mannosidase II is the most extensively studied GH38 enzyme, and it trims two α1,3 and α1,6‐linked terminal mannose residues from the substrate GlcNAcMan_5_GlcNAc_2_ during N‐glycosylation [18, 19].

In *S. cerevisiae*, Ams1 is transported from the cytosol into the vacuole (the equivalent of the lysosome in metazoans) through the cytoplasm‐to‐vacuole targeting (Cvt) pathway under both nutrient‐rich and starvation conditions [20, 21]. The Cvt pathway is a prototypic example of selective autophagy pathways where cargo molecules are recognized by specific autophagy receptors [22, 23]. In the Cvt pathway, Ams1 is recognized by the autophagy receptor Atg19 or its paralog Atg34 [21, 24, 25]. We have recently discovered a Cvt‐like selective autophagy pathway in the fission yeast *Schizosaccharomyces pombe* [26]. This pathway, termed Nbr1‐mediated vacuole targeting (NVT) pathway, uses the autophagy receptor Nbr1 to transport two cytosolic aminopeptidases Ape2 and Lap2 from the cytosol into the vacuole. Unlike the Cvt pathway in *S. cerevisiae*, the NVT pathway in *S. pombe* does not depend on the conventional autophagy machinery for cargo sequestration, but rather utilizes the endosomal sorting complex required for transport (ESCRT) machinery [26]. More recently, we found that *S. pombe* Ams1 is another cargo recognized by Nbr1 (manuscript in preparation). It is unclear how Ams1 is recognized by different autophagy receptors Atg19 and Nbr1 in the two yeasts. Structural information of Ams1 and its complex with autophagy receptors is crucial for answering this question. Preliminary crystallization of *S. cerevisiae* Ams1 (ScAms1) has been reported [27]. The structure of tetrameric ScAms1 was recently determined by cryo‐electron microscopy (cryo‐EM) at 6.3 Å [28], but the model was not of atomic resolution.

Here, we determined a cryo‐EM structure of *S. pombe* Ams1 (SpAms1) in the free state at 3.2 Å resolution. The structure was fortuitously obtained during analysis of its Nbr1 complex structure. This is the first atomic structure of tetrameric α‐mannosidase in the GH38 family. The structure reveals detailed interactions mediating tetramerization, the configuration of active site, as well as the structural divergence between SpAms1 and ScAms1.

## Results and Discussion

### Structure determination

**Fig. 1:**
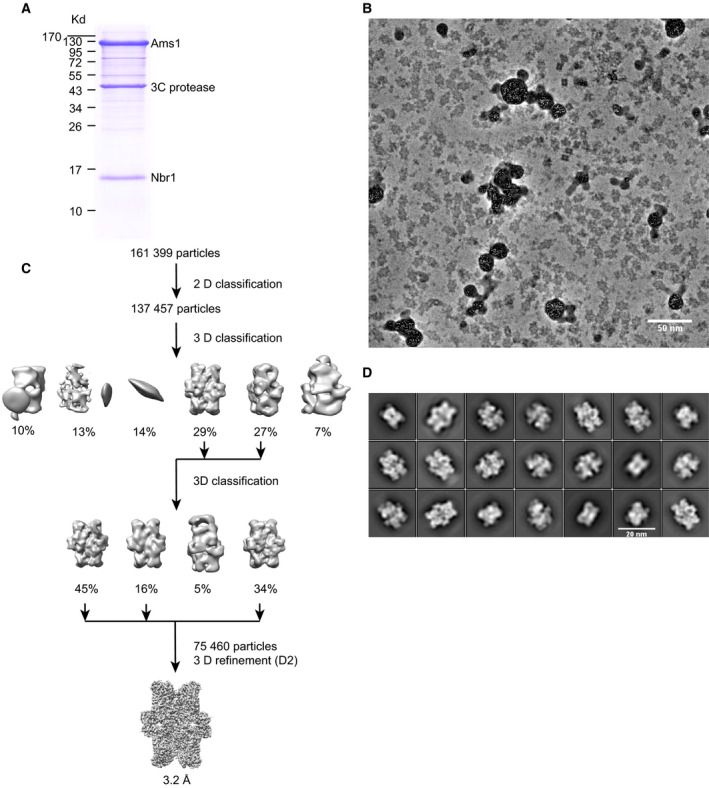
Cryo‐EM analysis of SpAms1. (A) SDS/PAGE analysis of copurified SpAms1‐Nbr1 complex. Positions of molecular weight markers are labeled on the left. 3C protease was used for cleaving between Nbr1 and its C‐terminal GFP tag so as to release the SpAms1‐Nbr1 complex from GFP‐Trap beads. (B) A representative cryo‐EM micrograph collected with a Volta phase plate. Scale bar = 50 nm. (C) Flowchart of image processing. (D) Representative 2D class averages. Scale bar = 20 nm.

To understand the structural basis of Nbr1 recognition of SpAms1, we attempted to determine their complex structure by cryo‐EM. A fragment of Nbr1 fused to GFP at the C terminus was co‐expressed with SpAms1 in *S. pombe* and bound to GFP‐Trap beads. Stoichiometric amounts of SpAms1 and Nbr1 proteins were copurified, indicating that they indeed formed a stable complex (Fig. 1A).

**Fig. 2:**
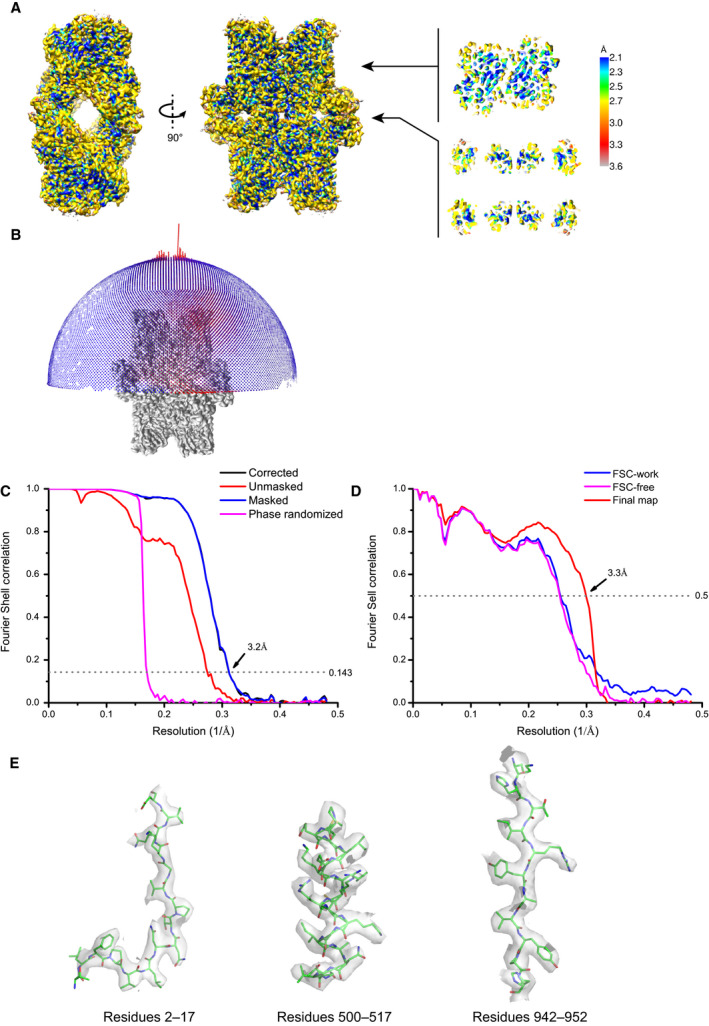
Quality of the cryo‐EM density map of SpAms1. (A) The cryo‐EM density map is colored by local resolution. Two cut‐through views are shown on the right. (B) Angular distribution of particles used in final reconstruction. Each cylinder represents number of particles in one particular orientation. (C) Fourier shell correlation (FSC) curves for the corrected (black), unmasked (red), masked (blue), and phase‐randomized (magenta) maps. The overall resolution is 3.2 Å according to the FSC = 0.143 criterion. (D) Model vs map FSC curves. Blue: FSC‐work between the re‐refined model and half‐map 1 against which the model was refined. Magenta: FSC‐free between the re‐refined model and half‐map 2 against which the model was not refined. Red: FSC between the refined structure and the postprocessed map. (E) Representative cryo‐EM densities fitted with the structural model.

**Fig. 3:**
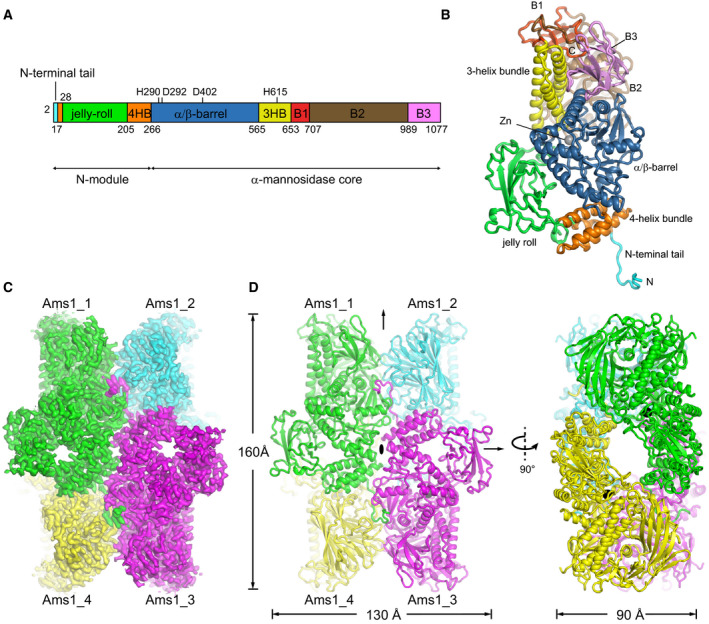
Cryo‐EM structure of the SpAms1 tetramer. (A) Domain diagram of SpAms1. The N‐terminal tail, a jelly‐roll domain, a 4‐helix bundle (4HB), an α/β‐barrel, a 3‐helix bundle (3HB), and the B1, B2, and B3 β‐sheet domains are color‐coded. The zinc‐binding residues are marked. (B) Ribbon representation of the SpAms1 monomer structure. Domains are colored as in A. The Zn ion is shown as a sphere. The structure is shown in the same view as subunit 1 in C. (C) Cryo‐EM density map of the SpAms1 tetramer. Subunits 1 to 4 are colored in green, cyan, purple, and yellow, respectively. (D) Ribbon representation of the SpAms1 tetramer structure in two orthogonal views. Three dyad axes are labeled.

**Fig. 4:**
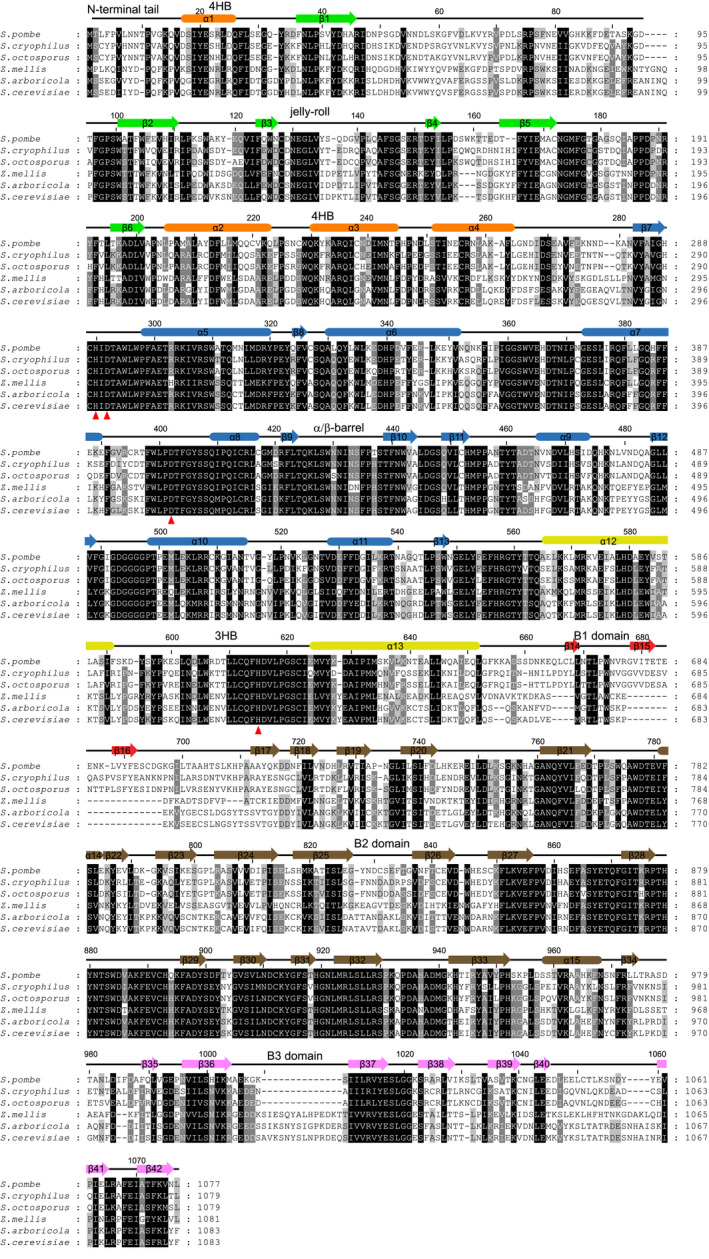
Multiple sequence alignment of Ams1 proteins from fission and budding yeasts. The sequences are from three fission yeast species (*Schizosaccharomyces pombe*, *Schizosaccharomyces cryophilus* and *Schizosaccharomyces octosporus*) and three budding yeast species (*Zygosaccharomyces mellis*, *Saccharomyces arboricola* and *Saccharomyces cerevisiae*). The residues conserved in at least 100%, 80%, and 60% of these sequences are shaded in black, gray, and light gray, respectively. The secondary structure elements observed in the cryo‐EM structure of SpAms1 are shown on the top and colored by domain. The Zn‐coordinating residues are denoted by red triangles at the bottom.

The vitrified specimen of SpAm1‐Nbr1 complex was prepared in holey carbon grids covered with a thin layer of carbon, which was critical for even distribution of particles in grid holes. Micrographs were collected in a Titan Krios 300 KV electron microscopy equipped with a Volta phase plate, a K2 Summit detector, and an energy filter (Fig. 1B). Following 2D and 3D classification (Fig. 1C,D), 75 460 particles were selected to reconstruct a density map with D2 symmetry at an overall resolution of 3.2 Å (Figs 2A–E and 3). The local resolution of the map reached 2.1 Å at the structure core (Fig. 2A), and side chains were discernable for most amino acid residues (Fig. 2E). An atomic model was built for the entire sequence of SpAms1 (residues 2–1077) except for the first methionine residue and the C‐terminal tag residues (Figs 3 and 4). To our disappointment, no density was left that can be assigned to Nbr1, suggesting that this structure represents SpAms1 in the free state. It is unlikely that Nbr1 was invisible in the density map due to its binding to a mobile loop of SpAms1, because all but one residues of SpAms1 are well structured. The copurified complex may have disassembled during cryo‐EM sample preparation, as macromolecules in thin aqueous films can be destabilized at the extensive solvent air interface [29]. Strategies to stabilize the complex will be tested to solve the complex structure.

### Structure of the SpAms1 tetramer

The structure reveals that SpAms1 forms a tetramer, like its homolog in *S. cerevisiae* [28]. Each subunit is sequentially composed of a jelly‐roll domain, a four‐helix bundle (4HB), an α/β‐barrel domain, a three‐helix bundle (3HB), and three predominantly β‐sheet domains (B1, B2, and B3) (Fig. 3A,B). The four‐helix bundle is built from two separated sequences flanking the jelly‐roll domain. In addition, a prominent 16‐residue N‐terminal tail projects from the structure core and mediates extensive inter‐subunit interactions.

**Fig. 5:**
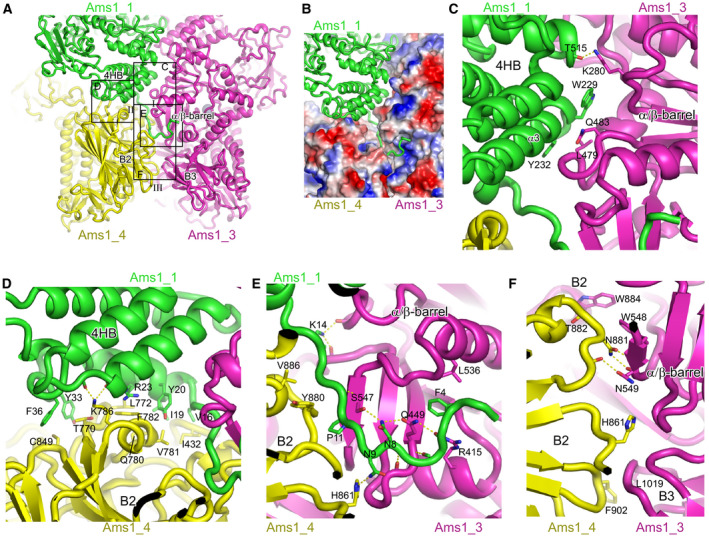
Tetramerization interfaces of SpAms1. (A) Three types of inter‐subunit interfaces among subunit 1 (green), 3 (magenta), and 4 (yellow). Boxed regions are shown in detail in C‐F. (B) Binding of the N‐terminal tail. Subunit 1 is represented as ribbons, and subunits 3 and 4 as electrostatic potential surfaces that are colored blue to red for positively to negatively charged regions. (C) Interface between subunits 1 and 3. Interacting residues are shown in sticks and hydrogen bonds are denoted by dashed lines. (D) Interface between subunits 1 and 4. (E) Interaction of the N‐terminal tail of subunit 1 with subunits 3 and 4. (F) Interface between subunits 3 and 4.

Four SpAms1 molecules, designated clockwise as subunit 1–4, assemble into a tetrameric structure with dimensions of 160 × 130 × 90 Å (Fig. 3C,D). The structure is of D2 symmetry and contains three dyad axes that are orthogonal to each other. Each subunit contacts the other three subunits, forming three types of inter‐subunit interface that are each present in four copies in the tetramer structure. Figure 5 illustrates a type I interface between subunits 1 and 3, a type II interface between subunits 1 and 4, and a type III interface between subunits 3 and 4. These three interfaces bury a solvent accessible surface area of 1125, 1069, and 657 Å^2^ per subunit, respectively. One set of interactions formed among subunits 1, 3, and 4 will be described (Fig. 5A).

The N‐terminal tail of subunit 1 adopts an extended conformation and inserts into a cleft formed between the α/β‐barrel domain of subunit 3 and the B2 domain of subunit 4 (Fig. 5B,E). The tail makes mainly van der Waals interactions with subunit 4 and a large number of potential hydrogen bonds with subunit 3. In addition, the phenyl ring of F4 is anchored in a hydrophobic pocket of subunit 3.

The 4HB of subunit 1 contacts the α/β‐barrel of subunit 3 primarily by van der Waals interactions (Fig. 5C). The aromatic rings of W229 and Y232, along with the backbone atoms of helix α3, constitute a flat surface contacting subunit 3. Mutation of the equivalent residue of W229 in ScAms1 disrupted the formation of tetramer [28], underscoring the importance of the interface. In addition, K280 of subunit 3 forms a hydrogen bond with the carbonyl oxygen of T515 of subunit 1.

The other side of the 4HB of subunit 1 contacts the B2 domain of subunit 4 (Fig. 5D). The interface is mainly composed of van der Waals interactions and involves V16, I19, Y20, R23, Y33, and F36 in subunit 1 and I432, V781, Q780, F782, T770, and C849 in subunit 4.

A type III interface is formed between the α/β‐barrel, B2 and B3 domains of subunit 3, and the B2 domain of subunit 4 (Fig. 5F). The tryptophan rings of W884 and W548 in subunit 3 constitute a hydrophobic depression that accommodates a protruding loop of subunit 4. The side chains of N881 and N549 of one subunit make hydrogen bonds with the backbone atoms of the other subunit. The interface is additionally stabilized by hydrophobic interactions between L1019 of subunit 3 and F902 of subunit 4.

### Comparison with other GH38 protein structures

**Fig. 6:**
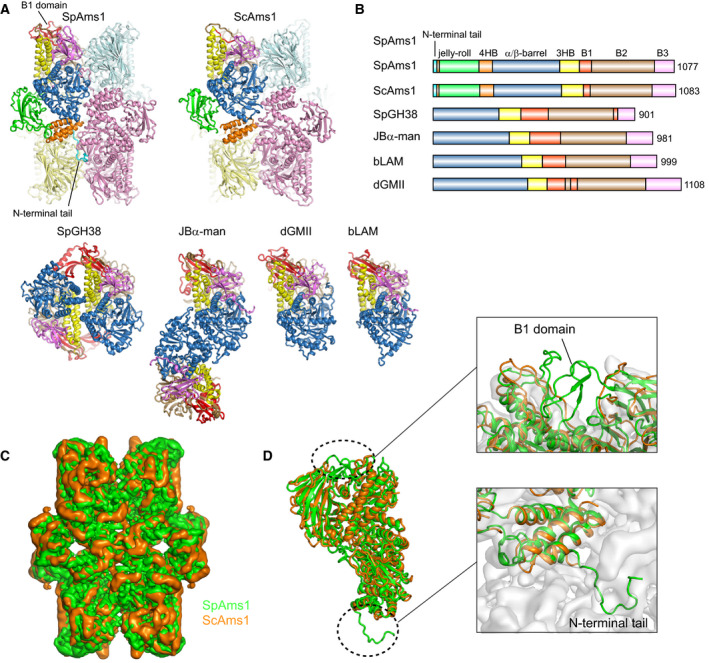
Structural comparison of SpAms1 with other GH38 α‐mannosidases. (A) Structural comparison of SpAms1, ScAms1 (PDB code: 5JM0), SpGH38 (2WYI), JBα‐man (6B9P), dGMII (3EJP), and bLAM (1O7D). The top‐left subunits of SpAms1 and ScAms1 tetramers, the right‐side subunits of SpGH38 and JBα‐man dimers, dGMII and bLAM are aligned to the same orientation. In SpAms1 and ScAms1 tetrameric structures, the top‐left subunits are colored by domain as in Fig. 1B and the other three subunits are colored palecyan, pink and paleyellow, respectively. All subunits of the other structures are colored by domain. (B) Domain organization diagrams of GH38 α‐mannosidases shown in A. (C) Superimposition of cryo‐EM density maps of SpAms1 (green) and ScAms1 (orange). (D) Alignment of the monomer structures of SpAms1 and ScAms1. The inserts are enlarged views of the B1 domain and the N‐terminal tail. The aligned structures are shown on the cryo‐EM map of ScAms1.

**Fig. 7:**
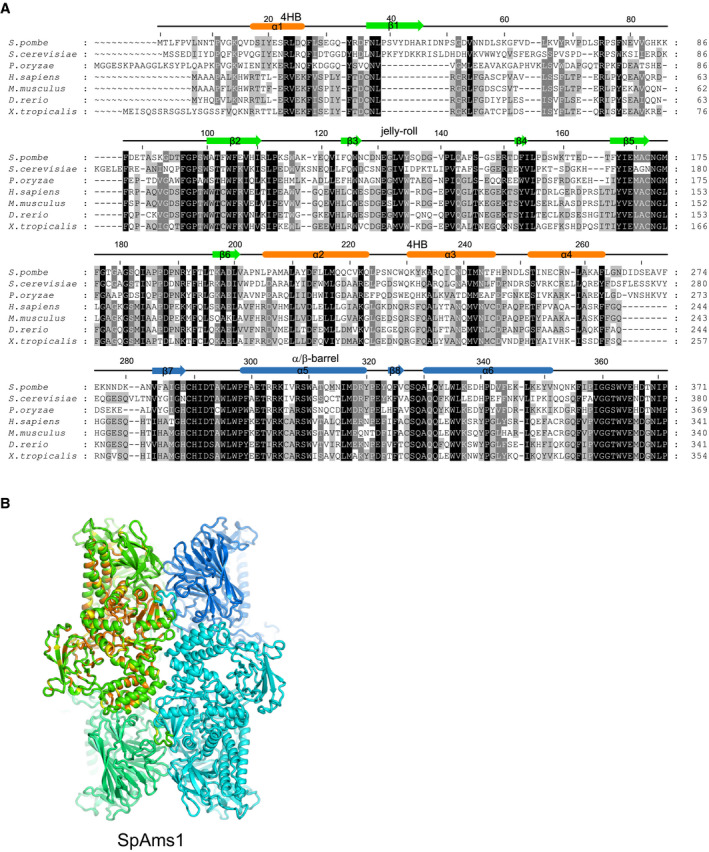
Homology between fungal Ams1 proteins and vertebrate MAN2C1 proteins. (A) Multiple sequence alignment of fungal Ams1 proteins and vertebrate homologs of MAN2C1. The sequences are from three fungal species (*S. pombe*, *S. cerevisiae*, the rice blast fungus *Pyricularia oryzae*) and four vertebrate species (*Homo sapiens*, *Mus musculus*, *Danio rerio,* and *Xenopus tropicalis*). The residues conserved in at least 100%, 80%, and 60% of these sequences are shaded in black, gray, and light gray, respectively. The secondary structure elements observed in the cryo‐EM structure of SpAms1 are shown on the top and colored by domain. Only the N‐terminal region of the alignment is displayed. (B) Residues conserved among Ams1 and MAN2C1 are mapped to the SpAms1 structure. The residues in subunit 1 with 100% and 80% conservation in the alignment shown in A are colored orange and yellow, respectively.

Structures have been determined for several α‐mannosidases in the GH38 family, including *Drosophila* Golgi α‐mannosidase II (dGMII), bovine lysosomal α‐mannosidase (bLAM), Jack bean α‐mannosidase (JBα‐man), bacterial *Streptococcus pyogenes* α‐mannosidase (SpGH38), and ScAms1 [18, 19, 28, 30, 31, 32] (Fig. 6A,B). All GH38 structures share a similar α‐mannosidase core, but ScAms1 and SpAms1 contain extra jelly‐roll and 4HB domains at the N terminus. The two N‐terminal domains are specific to the tetrameric GH38 enzymes determined so far and the 4HB domain also directly mediates tetramerization (Fig. 5). The other GH38 structures are either monomeric or dimeric (Fig. 6A). MAN2C1 and its homologs in vertebrates share considerable sequence conservation with fungal Ams1 in both the N‐terminal domains and the α‐mannosidase core (Fig. 7), suggesting that MAN2C1 proteins adopt a similar tetrameric structure as Ams1. The molecular size of a recombinant human MAN2C1 protein in solution was also consistent with a tetramer [33].

SpAms1 and ScAms1 share 49% sequence identity and 64% sequence similarity (Fig. 4). As expected, their structures can be well superimposed with a root mean standard deviation (rmsd) of 2.012 Å over 707 Cα atoms (Fig. 6C,D). However, the two structures display two notable differences. First, the N‐terminal tail of SpAms1, which is extensively involved in tetramerization, is absent in the ScAms1 structure [28]. ScAms1 also possesses an N‐terminal tail of similar size (Fig. 4). The tail was not modeled in the ScAms1 structure, perhaps due to the low resolution (6.3 Å) of the density map (Fig. 6D), or a lack of stable conformation. The first ten residues of the tail are quite divergent between SpAms1 and ScAms1 (Fig. 4). In particular, two SpAms1 residues, F4 and N8, engaging in inter‐subunit interactions, are not conserved in ScAms1 (Fig. 5E). Thus, the N‐terminal tail may adopt a different conformation in ScAms1. The second difference between the two structures lies in the B1 domain. The B1 domain folds into a well‐structured β‐barrel in SpAms1, but is largely missing in the ScAms1 structure (Fig. 6D). The amino acid sequence of the B1 domain is poorly conserved and greatly shortened in ScAms1, accounting for the difference (Fig. 4). These structural differences between ScAms1 and SpAms1 in the N‐terminal tail and the B1 domain may be relevant to how they are recognized by divergent selective autophagy receptors in *S. cerevisiae* and *S. pombe*.

### Active site of SpAms1

**Fig. 8:**
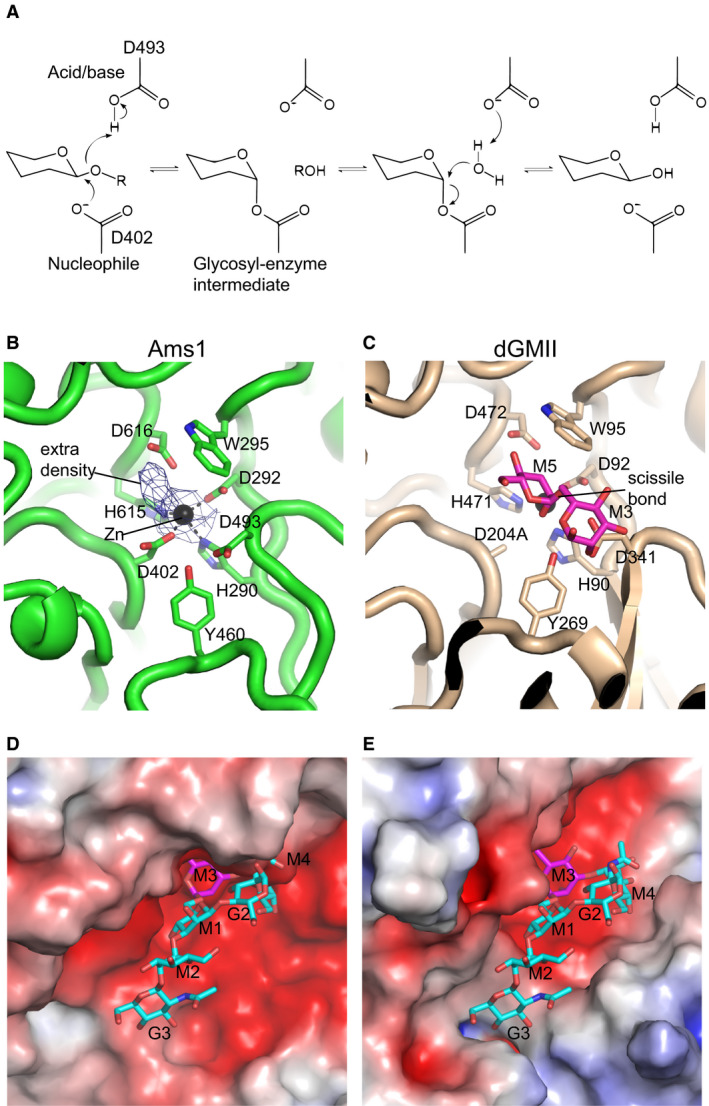
Active site and substrate‐binding pocket of SpAms1. (A) The retaining mechanism for enzymatic hydrolysis of glycosidic bond. The putative nucleophile and general acid/base of Ams1 are indicated. (B) Active site of SpAms1. Key residues for catalysis and substrate binding are shown in sticks. Cryo‐EM density is displayed for the Zn ion and an unmodeled molecule. (C) Active site of the D204A mutant of dGMII in complex with GlcNAcMan_5_GlcNAc (PDB code: 3CZN). G: GlcNAc, M: mannose. Only M5 and M3 mannose residues are shown in this view. The structure is aligned to the SpAms1 structure in A. (D‐E) Substrate‐binding pocket of SpAsm1 (D) and dGMII (E) shown in the same orientation as in B‐C. Proteins are represented as electrostatic potential surfaces that are colored red to blue for negatively to positively charged regions. The full substrate of dGMII is shown in E and modeled for SpAms1 in D. Carbon atoms are colored purple for M3 and M5 and cyan for other sugars.

The active site of SpAms1 is located in the α/β‐barrel and the 3‐helix bundle and is open to an internal channel with cross‐section dimensions of ~ 19 × 34 Å. The wide channel should allow oligosaccharide substrates to access the active site (Fig. 3D). As enzymes of glycoside hydrolase family 38 commonly contain a zinc ion in the active site [18], a strong density present in the active site of Ams1 was modeled as a zinc ion (Fig. 8B). The zinc ion is coordinated by four invariant residues H290, D292, D402, and H615. The zinc ion is additionally bound by an unmodeled density that may originate from small molecules, such as Tris. Tris molecules from buffer have been found to coordinate the zinc ion in crystal structures of unliganded dGMII and bLAM [18, 32].

Enzymatic hydrolysis of glycosidic bond requires a nucleophile and a general acid/base catalyst, which are commonly aspartate or glutamate residues, at the active site [34]. α‐Mannosidases in the GH38 family cleave terminal α‐linked mannose by a retaining mechanism (Fig. 8A), in which an acidic carboxylate protonates the glycosidic oxygen and the nucleophile attacks the anomeric carbon to form a covalent glycosyl‐enzyme intermediate. The glycosyl‐enzyme intermediate is then hydrolyzed by a water molecule that is activated by the now deprotonated carboxylate, releasing a product with the same stereochemistry as the substrate.

The active site of SpAms1 is compared with that of a nucleophile mutant of dGMII bound to its natural substrate GlcNAcMan_5_GlcNAc_2_ [19]. The active site of SpAms1 contains a set of highly conserved residues (Fig. 8B,C), suggesting that the mechanism of catalysis and substrate recognition at the −1 subsite are conserved between SpAms1 and dGMII. Based on the alignment, D402 and D493 appear to be the nucleophile and general acid/base catalyst, respectively. W295, Y460, and D616 likely function in substrate binding and stabilization of the transition state.

dGMII is highly specific on the substrate GlcNAcMan_5_GlcNAc_2_ and additionally recognizes the Man 4 (M4) and GlcNAc 3 (G3) residues of its substrate (Fig. 8E) [19]. Compared with dGMII, the substrate‐binding pocket outside the active site in SpAms1 is much wider, which may allow SpAms1 to accommodate diverse oligosaccharide substrates (Fig. 8D,E).

## Conclusion

We have determined a cryo‐EM structure for SpAms1, a tetrameric GH38 enzyme. The structure reveals the tetramerization interface and the active site at atomic resolution. Compared with the ScAms1 structure, SpAms1 contains unique structures at the N‐terminal tail and the B1 domain. The configuration of the active site and substrate‐binding pocket suggests that SpAms1 shares a conserved catalytic mechanism with other GH38 enzymes and may have wide substrate specificity.

## Materials and methods

### Fission yeast strain and plasmids

Genetic methods and composition of media are as described [35]. The sequence encoding SpAms1 fused at the C terminus with a Flag‐Flag‐His_6_ (FFH) tag was placed under the control of the *Pnmt1* promoter in a modified pDUAL vector [36], resulting in the plasmid pDB4788. The sequence encoding residues 53–180 of Nbr1 fused at the C terminus with two copies of human rhinovirus 3C protease cleavage site and a green fluorescent protein (GFP) was placed under the control of the *Pnmt1* promoter in a modified pDUAL vector, resulting in the plasmid pDB4789. pDB4788 and pDB4789 were transformed into the *S. pombe* strain LD259, resulting in the strain DY47304, whose genotype is *h^+^ ura4‐D18 his3‐D1 leu1‐32::Pnmt1‐ams1‐FFH(leu1^+^) ars1::Pnmt1‐nbr1(53‐180)‐2*×*3Csite‐GFP(ura4^+^)*.

### Purification of SpAms1‐Nbr1 complex from fission yeast

DY47304 was grown to mid‐log phase in EMM medium at 30 °C. About 2000 OD_600_ units of cells were harvested and washed once with water. Cells were lysed by grinding in liquid nitrogen. The resulting powder was mixed with lysis buffer (50 mm HEPES‐NaOH, pH 7.5, 150 mm NaCl, 1 mm EDTA, 1 mm dithiothreitol, 1 mm phenylmethylsulfonyl fluoride, 0.05% NP‐40, 10% glycerol, 1× Roche protease inhibitor cocktail). After centrifugal clarification, the cell lysate was incubated with GFP‐Trap Sepharose beads (ChromoTek, Planegg‐Martinsried, Germany) for 3 h at 4 °C. The beads were washed 4 times with wash buffer (50 mm HEPES‐NaOH, pH 7.5, 150 mm NaCl, 1 mm EDTA, 1 mm dithiothreitol, 0.05% NP‐40, 10% glycerol) and incubated with 100 μL of lysis buffer and 2 μg of 3C protease overnight at 4 °C. Protease‐released proteins were concentrated and buffer‐exchanged to storage buffer (50 mm Tris/HCl, pH 7.5, 150 mm NaCl, 5 mm MgCl_2_) by using an Amicon Ultra‐0.5 centrifugal filter with 30 kDa molecular weight cutoff (Millipore, Burlington, MA, USA).

### Cryo‐EM data collection

The vitrified specimen was prepared in a Vitrobot chamber (FEI) set to 100% humidity at 4 °C. Three microliters of sample (OD280 ~ 0.5) was incubated on a glow‐charged holey carbon grid (Quantifoil 300 mesh copper R1.2/1.3) covered with a home‐made continuous thin layer of carbon for 10 s, blotted for 5 s, and rapidly plunged into liquid ethane. Frozen grids were stored in liquid nitrogen.

Cryogenic samples were screened on a Talos F200C 200 kV electron microscope equipped with a Ceta camera (FEI). High‐resolution data were collected on a 300 kV Titan Krios electron microscope (FEI), which is equipped with a Volta phase plate (FEI), a K2 Summit detector and a GIF quantum energy filter (Gatan，Pleasanton, CA, USA). The energy filter was operated in a zero‐energy‐loss mode with a slit width of 20 eV. A total of 597 images were recorded with SerialEM [37]. Each image was composed of 32 frames and exposed for 8.4 s with an electron dose of 60 e·Å^−2^. The physical size of pixel was 1.04 Å. Defocus values ranged between −0.4 and −1.0 µm.

### Image processing

The recorded movie stacks were motion corrected by motioncor2 [38]. Parameters of contrast transfer function (CTF) were determined with Gctf [39]. Particles were automatically picked by gautomatch (http://www.mrc‐lmb.cam.ac.uk/kzhang/Gautomatch/). Further image processing was conducted with relion‐2.1 [40]. Particles were extracted with a box size of 240 pixels, downsized by fourfold and subjected to 2D and 3D classification with a mask of 200 Å diameter. The cryo‐EM density map of ScAms1 (EMD‐8166) was low‐pass filtered to 30 Å and used as the initial model for 3D classification [28]. After one round of 2D classification and two rounds of 3D classification, 75 460 particles from high‐resolution classes were selected for 3D auto‐refinement with imposed D2 symmetry. The final density map was postprocessed with correction of modulation transfer function of detector and B‐factor sharping. The overall resolution of the map was estimated with the criterion of gold‐standard Fourier shell correlation (FSC) of 0.143 [41]. Local resolution was calculated with resmap [42].

### Model building and refinement

A homology model of SpAms1 was generated by Phyre2 using the ScAms1 structure as template [43]. The model was adjusted and rebuilt in COOT [44] and refined in real space with secondary structure and geometry restraints by PHENIX [45]. The refinement statistics are summarized in Table 1. The structure validation statistics were reported by PHENIX. FSC curve between two maps was determined with relion_image_handle. To calculate FSC between model and map, the model was converted into a density map in Chimera, which was resampled to the grid of the map used for refinement. For cross‐validation, the model was re‐refined against half‐map 1. FSC‐work and FSC‐free curves were calculated for the re‐refined model map against half‐map 1 and half‐map 2 that was not used in refinement, respectively. Structural figures were prepared with pymol (Schrödinger, LLC) and Chimera [46]. Buried solvent accessible surface areas were computed with areaimol using a probe of 1.4 Å radius [47]. Nitrogen and oxygen atoms that are closer than 3.2 Å are considered to form hydrogen bonds.

**Table 1:** Statistics of data collection, structural refinement, and model validation.

Data collection	
EM equipment	FEI Titan Krios G2
Voltage (kV)	300
Detector	Gatan K2 summit
Energy filter	GIF Quantum Model 967 (20 eV slit)
Phase plate	Volta
Grid	Quantifoil Cu R1.2/1.3
Pixel size (Å)	1.04
Electron dose (e^−^·Å^−2^)	60
Defocus range (μm)	−0.4 to − 1.0
Map refinement	
Symmetry imposed	D2
Resolution (Å)	3.2
FSC threshold	0.143
Map‐sharpening *B* factor (Å^2^)	−116.9
Model composition	
Protein chains	4
Protein residues	4316
Zn ions	4
Structural refinement	
CC_mask	0.7654
CC_volume	0.7568
CC_peaks	0.7502
RMSD bonds (Å)	0.004
RMSD angles (°)	0.884
Validation	
Molprobity score	1.84
All‐atom clashscore	7.68
Rotamer outliers (%)	0.21
Ramachandran plot favored (%)	92.91
Ramachandran plot allowed (%)	7.09
Ramachandran plot outliers (%)	0

## Conflict of interest

The authors declare no conflict of interest.

## Author contributions

KY and LLD conceived and supervised the study; YYW prepared the sample; JZ determined the structure; JZ, LLD, and KY wrote the manuscript.

## Data Availability

The cryo‐EM density map and coordinates have been deposited to Electron Microscopy Data Bank (EMDB) and Protein Data Bank (PDB) under accession numbers EMD‐30021 and 6LZ1.
